# Trajectories of wellbeing in people who live with gamblers experiencing a gambling problem: An 18-year longitudinal analysis of the Household, Income and Labour Dynamics in Australia (HILDA) survey

**DOI:** 10.1371/journal.pone.0281099

**Published:** 2023-01-27

**Authors:** Catherine Tulloch, Matthew Browne, Nerilee Hing, Matthew Rockloff, Margo Hilbrecht

**Affiliations:** 1 Experimental Gambling Research Laboratory, School of Health, Medical and Applied Sciences, Central Queensland University, Sydney, New South Wales, Australia; 2 Experimental Gambling Research Laboratory, School of Health, Medical and Applied Sciences, Central Queensland University, Bundaberg, Queensland, Australia; 3 The Vanier Institute of the Family, Ontario, Canada; 4 The Department of Recreation & Leisure Studies, University of Waterloo, Ontario, Canada; Sheffield Hallam University, UNITED KINGDOM

## Abstract

In cross-sectional gambling studies, friends, family, and others close to those experiencing gambling problems (concerned significant others ‘CSOs’) tend to report detriments to their quality of life. To date, however, there have been no large, population-based longitudinal studies examining the health and wellbeing of CSOs. We analyse longitudinal data from the Household, Income and Labour Dynamics in Australia (HILDA) survey to examine the 18-year trajectories of general, social, health and financial wellbeing of household CSOs (n = 477) and compare these to those without a gambling problem in the household (n = 13,661). CSOs reported significantly worse long-term wellbeing than non-CSOs in their satisfaction with life, number of life stressors, and social, health and financial wellbeing. However, both social and financial wellbeing showed a temporal effect, declining significantly for CSOs at times closer to the exposure to the gambling problem. This finding suggests a causal link between living in a household with a person with a gambling problem and decreased CSO social and financial wellbeing. Policy responses, such as additional social and financial support, could be considered to assist CSOs impacted by another person’s gambling problem.

## Introduction

The impacts of gambling-related harm on the health and wellbeing of close family and friends of people who gamble (‘close significant others’, CSO’s) contribute to the public health burden associated with gambling problems [1]. Harm attributable to gambling can extend to CSOs across multiple areas of their lives, with the most commonly experienced being impacts to their psychological, social/relational, and financial wellbeing [2–4]. Because an ongoing gambling problem requires substantial financial outlay, financial impacts are the most commonly experienced issue [2, 3, 5, 6]. Financial stress is not only associated with a lack of money for bills and essential needs and a reduction in spending money, but also leads to increased stress and conflict within relationships [6]. Relationship harms can range from reduced time spent together [6] through to increased conflict [7–9] and domestic and family violence [10]. The CSO’s relationships with others can also be impacted. For example, they can experience a reduced social life [11, 12], become estranged or distanced from family and friends [13], and experience social rejection [14] and isolation [12]. Impacts to psychological wellbeing are commonly associated with CSOs and include emotional or psychological distress [e.g., 3, 15–17], anger and feelings of guilt [18], symptoms of anxiety and depression [18–20] and lower wellbeing and satisfaction with life [21]. These can then lead to physical health problems such as headaches [18, 22, 23] and reduced sleep [24, 25].

However, many of these relationships are bi-directional. For example, gambling can be used to try to improve finances [26]; where a household is struggling financially, a household member may attempt to solve this problem by gambling. For other people, gambling may have begun as a social activity; for instance couples might have started visiting gambling venues together as a response to feeling socially isolated [27]. Alternatively, gambling may be used as an escape, as a way to cope with or avoid negative emotions, including those associated with problems or stresses being experienced by others close to the gambler [28]. Therefore, a fundamental limitation to many CSO studies is their cross-sectional nature, as these wellbeing decrements may have predated exposure to the gambling problem. Furthermore, most of the studies mentioned above deal with self-nominated symptoms as described by CSOs. Thus, any reported harms might be over-attributed to the gambling rather than other causes, be exaggerated due to negative attitudes towards gambling, and/or conceivably not result in a significant impact to a CSO’s overall health and wellbeing. Overall, rather than a friend or family member’s gambling directly leading to health and wellbeing decrements in CSOs, the gambling problems may be a feature of those groups vulnerable to both poorer health and wellbeing outcomes and gambling problems, such as those experiencing economic deprivation [29–31]. Thus, the gambling may to some degree be a symptom rather than an underlying cause of such health or wellbeing deficits. If this alternative explanation is true, then one would expect that any observed decrement to wellbeing in CSOs should precede the reported gambling problems.

There are very few quantitative studies exploring the impact of gambling on CSOs longitudinally. One short-term study examined health and wellbeing factors associated with being close to someone with a gambling problem, following up a year later [15]. At the initial assessment, CSOs reported financial impacts, relationship problems, poor mental health, risky alcohol use, and a lack of social support (someone to talk to or help with practical issues). A year later, participants who were still defined as CSOs were compared to those who were not (ex-CSOs). Ex-CSOs reported fewer arguments and separations, improved mental health and fewer financial problems than in the first assessment; however, there were no differences in self-reported health or alcohol use. Another study followed children of parent/s experiencing a gambling problem [32], and found depressive symptoms increasing between mid-adolescence and early adulthood. Both these studies explored CSOs’ wellbeing after they had been exposed to problem gambling, so conclusions cannot be made about whether the ongoing problems were legacy harms attributable to gambling [6, 33], or ongoing longer-term co-morbid issues.

The present study seeks to understand whether gambling is a direct causal factor in wellbeing outcomes for household members of individuals experiencing a gambling problem. People who co-habit with a person experiencing a gambling problem are a key subset of the broader group of CSOs. They tend to have a very close relationship with the gambler, sharing household and financial responsibilities, and therefore tend to be the most acutely affected. We analyse longitudinal data from the Household, Income and Labour Dynamics in Australia (HILDA) survey, a large-scale population study, to explore the trajectory of wellbeing factors in CSOs. Using data from 2018, the most recent to assess gambling problems, the present study identifies household CSOs, that is, those people living with another person experiencing moderate or severe gambling problems at that time (i.e., 2018). We then examine the trajectory of the CSOs’ overall, social, health and financial wellbeing variables over the preceding 17 years and compare these to respondents who were identified as non-CSOs in 2018 as a non-experimental control group. Unfortunately, data on household gambling problems are not available for every year; however, gamblers are known to transition in and out of experiencing problems [34] and, as such, the probability of negative impacts from gambling should, on average, progressively reduce over the preceding years. In contrast, we would expect stable socio-economic factors that drive both exposure to gambling problems and CSO wellbeing outcomes to be relatively constant, and not to be time-synchronised to the observed gambling problems. Since these effects should not show a time-dependent gradient, analysis of the retrospective time-course of CSOs’ wellbeing can help distinguish consequences that are a direct outcome of the gambling, as opposed to being due to shared risk factors.

A key assumption is that the CSOs in this study were unlikely to have been exposed to gambling problems for the preceding 17 years. Their level of exposure involves two factors: how long the gambler has experienced a gambling problem, and how long the CSO has had an active relationship with that person. Overall, the bulk of evidence supports that more serious gambling problems may fluctuate over reasonably short periods of time [34]. While, Billi et al. [35] found severe gambling problems to be relatively stable over a 4-year period, more recent longitudinal studies have found episodes of problem gambling to last around 1 year, and unrelenting problems across 4 or 5 years to be relatively uncommon [36–39]. In looking specifically at CSOs, the evidence is more limited. In Riley et al.’s [40] qualitative study of partners of non-treatment-seeking gamblers, the mean length of the relationship between the couples was close to 10 years. Almost all participants recalled a time in their relationship when their partner’s gambling was not a problem. Another study of 50 family members of gamblers found that around 30% of the participants had been CSOs for less than three years [13]. Svensson et al. [15]’s population-based study found that just over half (54.7%) continued to be impacted a year later. While in some cases the gambling problem itself resolves, in other instances, CSOs may remove or distance themselves from the person with the gambling problem. This ‘withdrawal’ commonly occurs and is potentially a helpful coping method [41]. Separation and divorce rates are also high for individuals experiencing gambling problems, with those experiencing a severe problem being over twice as likely to be divorced than the general population [42]. Given this evidence, the current study is based on the relatively safe assumption that a household CSO is most likely to be experiencing negative impacts at the time gambling problems are identified in their household, and that, to the extent gambling is causing impacts to wellbeing, the expected impacts should decrease at increasingly distal times. Alternatively, if mean decrements to wellbeing are due to pre-existing long-term factors (e.g., economic deprivation), there should be no significant differences between the gradients (compared to households without a gambling problem) with respect to increasing time from the gambling problem.

In sum, this study examines a range of social, health and financial wellbeing factors up to and including the time at which the CSO is known to be exposed to another person’s gambling problem. We examine retrospective trajectories of wellbeing in CSOs impacted by gambling problems in 2018 compared to people without a gambling problem in the household. We expect that social, health and financial wellbeing are primarily outcomes of exposure to the gambling problem (rather than other causes or confounds that were measured), and accordingly hypothesise that CSO wellbeing should decline closer to a known time when they are impacted by the gambling problem. Since data on CSO status was also available in the 2015 wave, we also check assumptions against this data, examining the proportion of affected respondents who were also household CSOs three years prior.

## Methods

### Data source

The Household Income and Labour Dynamics in Australia Survey [HILDA; 43] is a longitudinal Australian survey that collects economic, social, health and demographic information. The study began in 2001 with an initial sample of 7,682 Australian households selected via a stratified three-stage cluster design, expanding to include new household members as household configurations changed. The sample was topped up with an extra 2,153 households in 2011, and by 2018, there were 9,639 responding households, comprising 23,237 people. Further details are available in Summerfield et al. [44], and Watson and Wooden [45]. Participants aged 15 and over were asked to annually participate in a face-to-face interview that included questions about subjective wellbeing. They also privately completed a paper-based questionnaire, which consisted of gambling-related questions and questions probing health, social support, community participation, financial stressors, and life events.

### Design and participants

The current study is a prospective cohort study. We used linear mixed models to examine 18-year trajectories of general, health, social and financial wellbeing outcome variables associated with those identified as CSOs or non-CSOs in 2018. The sample of interest for this study was participants aged 15 years or over (adults) who lived in households where all individuals completed the Problem Gambling Severity Index [PGSI; 46] in 2018. This enabled them to be categorised as CSOs or non-CSOs (as described in measures).

### Measures

#### Identifying CSOs

First, gambling problems were identified using the Problem Gambling Severity Index [PGSI; 46] as measured in the 2018 HILDA wave. The PGSI is a well-validated and commonly used measure of problematic gambling behaviour and its consequences over the past 12 months [47]. All household members were asked to complete the PGSI in relation to their own gambling. The 9-item questionnaire includes questions such as “How often have you bet more than you could really afford to lose?” which are rated on a four-point scale from 0 (never) to 3 (almost always). The total summed scores range between 0 and 27, by which respondents are classified as non-problem gamblers or non-gamblers (total score of 0), low-risk gamblers (1 to 2), moderate-risk gamblers (3 to 7), or problem gamblers (8+).

**Fig 1:**
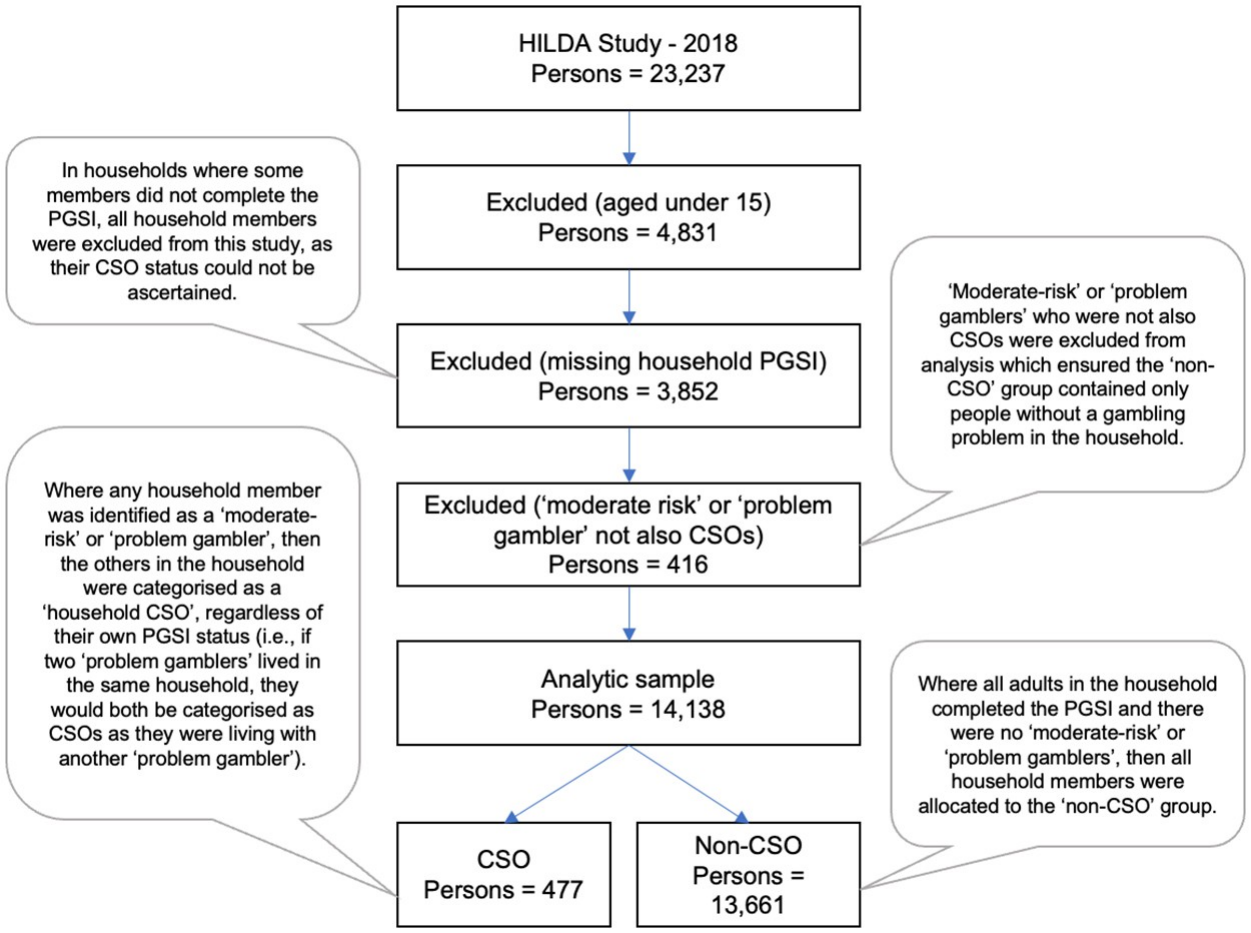
Primary sample selection, HILDA 2018.

The study then identified all the household members of participants who had been classified by their completed PGSI as either a ‘moderate-risk’ or ‘problem’ gambler. These participants were classified as CSOs as they lived with someone experiencing a gambling problem. Fig 1 describes the sample selection process used to identify CSOs and non-CSOs.

There were no significant sex differences between the excluded (missing household PGSI) group and the analytic sample. However, the mean age was significantly lower (*M* = 41.7 years, *SD* = 19.9) compared to the analytic sample (*M* = 46.6 years, *SD* = 18.9); Welch (5884.70) = 190.40, *p* <.01).

HILDA collected data on gambling behaviours only in 2015 and 2018. For CSOs identified in 2018, a similar categorisation was conducted in 2015 to assess the proportion of CSOs who were also household CSOs three years prior. Around one-quarter of the sample (25.2%) could not be categorised in 2015 as they, or their other household members, did not complete the PGSI in that wave.

#### Outcome variables

*Health*. Health state utility was measured by the SF-6D. This health index health state score is derived from the SF-36 [48], a measure of functional health and wellbeing which has been validated for use in Australian populations [49]. Scores range from 0 (worst health state) to 1 (best health state). HILDA calculates these utility values using Australian weights [50, 51]. The SF-36 was available across all 18 years.

*Social support*. The HILDA social support scale is used to assess an individual’s perceived social support [52–54]. The 10 questions include “I often need help from other people but can’t get it”, “I don’t have anyone that I can confide in”, and “When I need someone to help me out, I can usually find someone”. Responses are rated on a seven-point scale from 1 (strongly disagree) to 7 (strongly agree). Negatively worded items were reverse-scored, and responses were summed to create a scale. Higher scores represent a higher level of perceived social support. Cronbach’s Alpha for the scale was α = .85, indicating adequate internal reliability. HILDA included this scale in its current form from 2003 onwards.

*Community participation*. Community participation was assessed using a 12-item measure adapted for use in HILDA from the Australian Community Participation Questionnaire [55]. Questions include “Attend events that bring people together such as fetes, shows, festivals or other community events”, “Get involved in activities for a union, political party, or groups that is for or against something” and “Chat with your neighbours” and cover three broad categories of participation: civic engagement, political participation, and informal social connectedness. Responses are rated on a six-point scale from 1 (never) to 6 (very often). Higher scores represent greater community participation. Scale reliability for this study was Cronbach’s Alpha, α = .80. HILDA measured community participation in 2006, 2010, 2014 and 2018.

*Financial stressors*. Indicators of financial stress were assessed via seven yes/no questions. These were “could not pay electricity, gas or telephone bills on time”, “could not pay the mortgage or rent on time”, “pawned or sold something”, “went without meals”, “was unable to heat home”, “asked for financial help from friends and family” and “asked for help from welfare/community organisation”. The number of positive responses was summed with a higher number indicative of more financial stressors. Financial stressors were measured across all years except 2010.

*Personal stressors*. HILDA assesses various life events, both positive and negative. Personal stressors were assessed from the following 14 life events “separated from spouse”, “serious personal injury/illness”, “serious injury/illness to family member”, “death of a spouse or child”, “death of a close relative/family member”, “death of a close friend”, “victim of physical violence”, “victim of a property crime”, “detained in jail”, “close family member detained in jail”, “retired from the workforce”, “fired or made redundant”, “changed jobs” and “major worsening in finances”. Participants indicated yes or no to these events occurring in the previous 12-months, and the number of positive responses was summed. Personal stressors were measured across all years except 2001. Deviant personal stressors were those associated with violence and crime. They included “victim of physical violence”, “victim of a property crime”, “detained in jail”, and “close family member detained in jail”.

*Subjective wellbeing (SWB)*. A single item life satisfaction question probed global wellbeing: “All things considered, how satisfied are you with your life?”. Specific wellbeing domains were assessed via questions that asked people to rate how “satisfied or dissatisfied you are with some of the things happening in your life”. Participants were then shown a list which included “Your financial situation”, “Feeling part of your local community”, “The neighbourhood in which you live” and “Your health”. All responses were rated on an 11-point scale from 0 (totally dissatisfied) to 10 (totally satisfied). SWB was measured across all years.

### Covariates

A range of socio-demographic variables associated with gambling problems [56] were included in the study. These included age, sex, education, and household income.

### Statistical analysis

Participants’ characteristics in 2018 were analysed using descriptive analyses in IBM SPSS Statistics for Windows, Version 27 [57]. These analyses detailed the total sample, CSOs and non-CSO groups, with differences assessed using chi-square tests and t-tests. Longitudinal data (2001–2018) was then analysed utilising the R statistical programming environment [58]. This analysis was used to examine the preceding years’ responses, allowing the identification of any CSO trajectories different from non-CSOs. To maximise sample size and power, the study did not exclude participants who were missing from some of the previous waves (e.g., did not complete the questionnaire that year, or began participation in a later year due to joining a ‘HILDA household’ or being associated with the 2011 top up sample). A linear mixed model was fit using the REML criterion. Repeated measures were included within-subjects, across years of assessment. Each model included fixed effects predicting each outcome from CSO status, demographic control variables (sex, age, education, and income), plus a linear effect for the year relative to 2018: the year in which the assessment of CSO status occurred, and an interaction term between year and CSO status. Thus, a significant beta coefficient for CSO indicates an overall difference in the outcome over all years that is not dependent on recency with respect to observed CSO status. A significant main effect for year indicates a systematic change in the outcome over time that is not moderated by CSO status, likely due to age or cohort effects. However, a significant interaction between year and CSO status indicates that the difference in the outcomes between CSOs and non-CSOs was moderated by recency to the time of positive CSO identification in 2018. Our original analysis intention was to include not only a random intercept for each participant but also a random slope for year. However, this model presented numerical convergence issues for several outcome variables. Therefore, we were obliged not to include this effect. From informal comparison of models that did converge, our opinion is that this slight model misspecification should not present major issues for inference regarding the main analysis goals. The linear mixed model design considers the clustering of observations within persons; however, in some cases, multiple CSOs reside in a single household and, as such, are linked to the same gambler. Therefore, there may be some potential clustering of outcomes within households, technically violating the assumption of independence. Convergence issues associated with introducing a further random factor prevented this inclusion to account for this co-variance. To increase beta comparability, all numeric and ordinal variables (both IVs and DVs) were scaled (mean = 0, SD = 1). Natural binary variables like sex were not scaled. A *p*-value of <.05 was considered significant.

### Ethics

The HILDA Study has been conducted annually since 2001, following the University of Melbourne’s ethics guidelines. Ethics approval for data collection was granted by the Human Research Ethics Committee of the University of Melbourne Ethics (#1955879) and updated annually. This paper uses de-identified unit record data, so consent was not required for this study, and approval for secondary analysis was granted by Central Queensland University Human Research Ethics Committee (#22878).

## Results

Characteristics of participants in 2018 are described in Table 1. Of the total sample, 3.5% were identified as CSOs (i.e., they lived in a household where the PGSI has classified another member as a moderate-risk or problem gambler). CSOs were significantly more likely to be female, be low or moderate risk or problem gamblers themselves, and be younger and less well-educated than non-CSOs. Of the CSOs in 2018 who could be categorised in 2015, around half (49.9%) were also CSOs three years previously (50.1% were not CSOs).

**Table 1:** Participant characteristics in 2018 by total sample and CSO status.

	Total Sample		CSO		Non-CSO		
	N	*%*	N	*%*	N	*%*	*p*
Sex							
Male	6686	47.3	199	41.7	6487	47.5	.013
Female	7452	52.7	278	58.3	7174	52.5	
Marital Status							
Never married	3051	21.6	115	24.1	2936	21.5	.006
Married/cohabiting	6582	60.7	298	62.5	8284	60.6	
Separated/divorced	1776	12.6	55	11.5	1721	12.6	
Widowed	727	5.1	9	1.9	718	5.3	
Education							
did not complete high school	3278	23.2	146	30.6	3132	22.9	<.001
completed high school	2154	15.2	80	16.8	2074	15.2	
completed further education	8700	61.6	251	52.6	8449	61.9	
Employment							
part time employment	2944	20.8	102	21.4	2842	20.8	.034
full time employment	6117	43.3	211	44.3	5906	43.3	
unemployed	452	3.2	23	4.8	429	3.1	
retired	2982	211	78	16.4	2904	21.3	
other	1631	11.5	62	13	1569	11.5	
Income Band							
Under $20,000	469	3.4	7	1.5	462	3.4	<.001
$20,000–$39,999	2053	14.7	44	9.4	2009	14.9	
$40,000–$59,999	1881	13.5	54	11.5	1827	13.5	
$60,000–$79,999	1487	10.7	56	12	1431	10.6	
$80,000–$99,999	144	10.3	55	11.8	1389	10.3	
$100,000–$124,999	1700	12.2	83	17.7	1617	12	
$125,000–$149,999	1366	9.8	46	9.8	1320	9.8	
$150,000–$199,999	1818	13	61	13	1757	13	
$200,000 or more	1736	12.4	62	13.2	1674	12.4	
PGSI Category*							
non-problem gambler	13142	95.6	355	76.7	12787	96.2	<.001
low risk gambler	538	3.9	37	8	501	3.8	
moderate risk gambler	63	0.5	63	13.6	0	0	
problem gambler	8	0.1	8	1.7	0	0	
	Mean	*SD*	Mean	*SD*	Mean	*SD*	*p*
Age	*N* = 14138		*n* = 477		*n* = 13661		
	46.6	18.9	43.5	18.5	46.7	18.9	<.001
Household debt	$420,405	$221,180	$249,728	$468,019	$220,184	$418,629	0.131

* Problem- and moderate-risk gamblers were excluded unless they resided with another problem- and moderate-risk gambler and were therefore categorised as a CSO.

**Fig 2:**
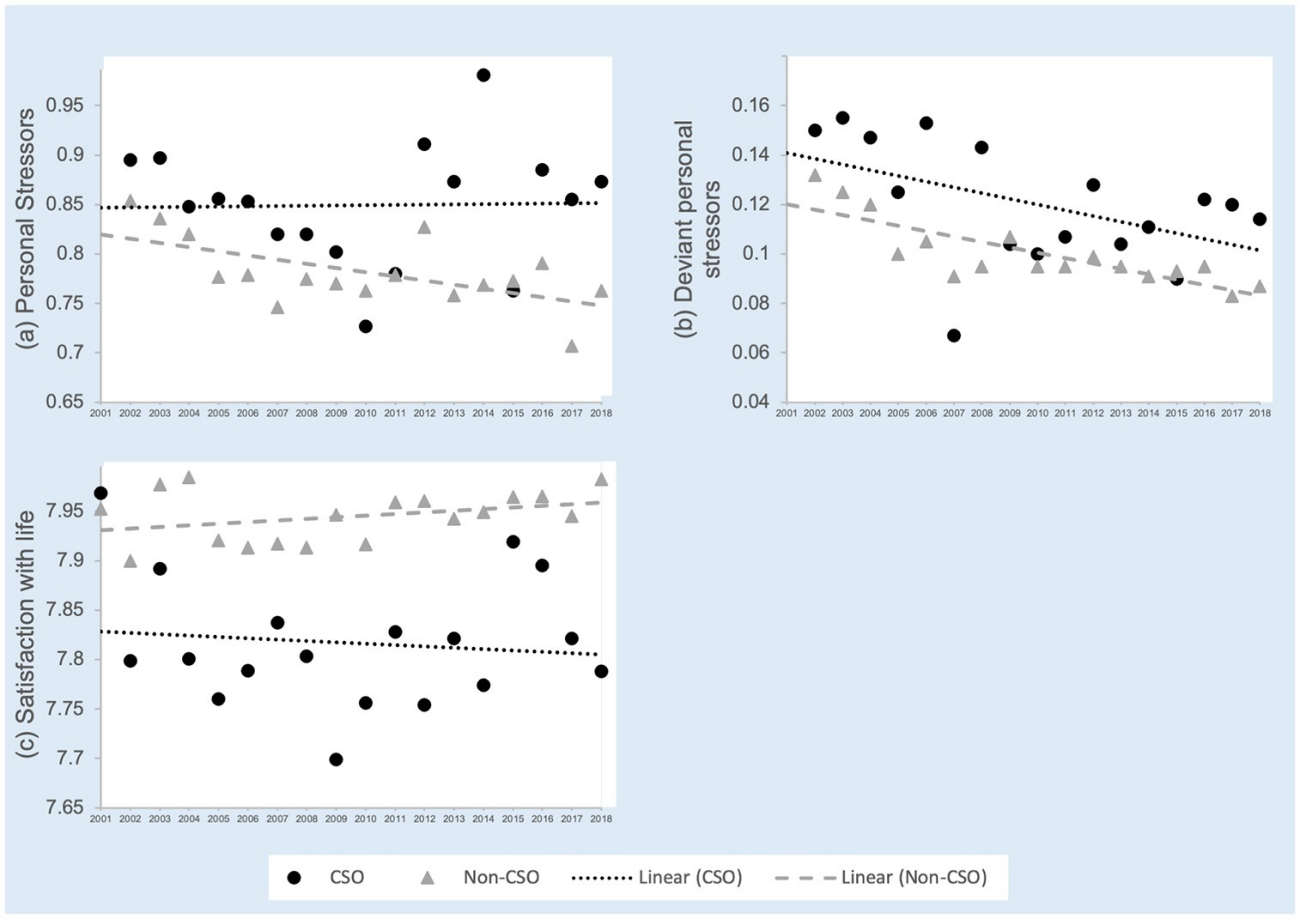
a-c. The trajectories of overall wellbeing for CSO and non-CSOs.

**Fig 3:**
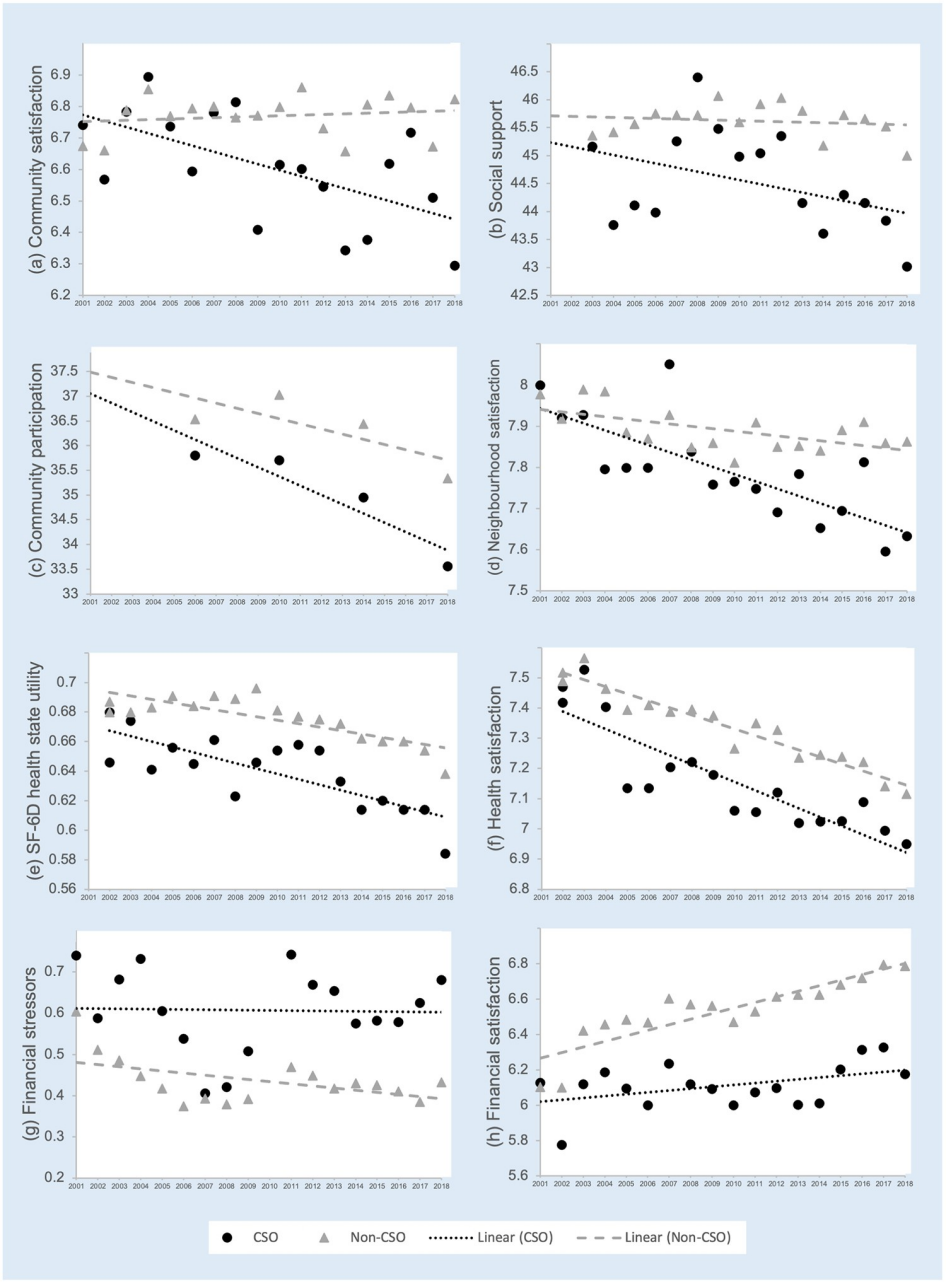
a-h. The trajectories of social, health and financial wellbeing for CSO and non-CSOs.

The trajectories of overall wellbeing, the average number of personal stressors, deviant personal stressors, and mean life satisfaction scores for CSO and non-CSOs are illustrated in Fig 2 (panels a—c). Fig 3 shows the trajectories associated with social (panels a—d), health (panels e—f) and financial (panels g—h) wellbeing. These figures identify the means for CSOs and non-CSOs for each year of data and the trendlines for each group.

### General wellbeing

The analyses shown in Table 2 indicate that overall, CSOs tend to have a significantly greater number of personal and deviant personal stressors, and lower satisfaction with life than non-CSOs across the previous years. There were no significant differences found between CSOs and non-CSOs in the slope of the trajectories over that time.

**Table 2:** Generalised linear mixed-effects model results for overall wellbeing variables.

Overall wellbeing DVs												
	Personal Stressors				Life Satisfaction				Deviant Personal Stressors			
Fixed Effects	Estimate	*SE*	*t*	*p*	Estimate	*SE*	*t*	*p*	Estimate	*SE*	*t*	*p*
(Intercept)	0.01	0.01	1.18	.239	0.02	0.01	1.98	.048	0.07	0.01	11.79	<.001
Year	0.03	0.00	8.30	<.001	0.01	0.00	3.47	.001	0.05	0.00	14.99	<.001
CSO—Yes	0.09	0.03	3.20	.001	-0.11	0.04	-3.02	.003	0.07	0.03	2.67	.008
Female	0.02	0.01	2.25	.025	0.02	0.01	1.67	.095	-0.05	0.01	-6.45	<.001
Age	-0.02	0.01	-4.63	<.001	0.05	0.01	7.06	<.001	-0.11	0.00	-25.20	<.001
Education	0.02	0.00	4.41	<.001	-0.05	0.01	-7.92	<.001	0.01	0.00	2.77	.006
Household Income	-0.11	0.01	-22.52	<.001	0.10	0.01	14.45	<.001	-0.07	0.00	-16.35	<.001
Year x CSO	-0.03	0.02	-1.44	.150	0.01	0.02	0.42	.671	0.01	0.02	0.26	.795
**Random Effects**	St. Dev	Correlation			St. Dev	Correlation			St. Dev	Correlation		
(Intercept)	0.42	0.07			0.68	0.05			0.34	0.24		
Year	0.14				0.22				0.16			
Residual	0.90				0.69				0.93			
Parameter estimates (phi)	0.17				0.15				0.13			
Observations	152900				168641				152754			
Groups	13910				13948				13910			

Note: all numeric and ordinal variables, both IVs and DVs (except those already binary) were scaled (m = 0, SD = 1). Correlation structure AR (1).

### Social wellbeing

CSOs reported significantly worse social wellbeing across all social DVs than non-CSOs (Table 3). The trajectories are similar for CSOs and non-CSOs for community participation and satisfaction with their neighbourhood. However, the trajectory of their satisfaction with the community and their perceived social support significantly declines with recency for CSOs compared to non-CSOs. That is, their social wellbeing in these areas becomes worse closer to the point at which the CSOs were identified as being close to someone with a gambling problem (2018).

**Table 3:** Generalised linear mixed-effects model results for social wellbeing variables.

	Social DVs															
	Community Participation Scale				Satisfaction with Community				Social Support Scale				Satisfaction with Neighbourhood			
Fixed Effects	Estimate	*SE*	*t*	*p*	Estimate	*SE*	*t*	*p*	Estimate	*SE*	*t*	*p*				
(Intercept)	-0.19	0.01	-16.75	<.001	-0.06	0.01	-7.09	<.001	-0.09	0.01	-9.30	<.001	-0.02	0.01	-2.48	.013
Year	0.03	0.00	7.44	<.001	-0.03	0.00	-7.59	<.001	0.02	0.00	4.92	<.001	0.01	0.00	1.89	.059
CSO—Yes	-0.13	0.05	-2.75	.006	-0.08	0.04	-2.31	.021	-0.14	0.04	-3.60	<.001	-0.07	0.04	-2.08	.038
Female	0.31	0.01	21.53	<.001	0.06	0.01	4.59	<.001	0.19	0.01	14.39	<.001	0.03	0.01	2.21	.027
Age	0.26	0.01	33.35	<.001	0.16	0.01	24.62	<.001	0.02	0.01	2.21	.027	0.13	0.01	20.07	<.001
Education	0.12	0.01	15.34	<.001	-0.01	0.01	-0.90	.367	0.02	0.01	3.11	.002	-0.02	0.01	-2.54	.011
Household Income	0.10	0.01	12.34	<.001	0.09	0.01	14.21	<.001	0.15	0.01	20.74	<.001	0.11	0.01	17.14	<.001
Year x CSO	0.03	0.02	1.27	.204	0.05	0.02	2.41	.016	0.05	0.02	2.48	.013	0.03	0.02	1.61	.108
**Random Effects**	St. Dev	Correlation			St. Dev	Correlation			St. Dev	Correlation			St. Dev	Correlation		
(Intercept)	0.74	-0.01			0.65	0.03			0.73	-0.04			0.61	0.03		
Year	0.16				0.24				0.23				0.22			
Residual	0.57				0.71				0.60				0.76			
Parameter estimates (phi)	0.09				0.17				0.15				0.21			
Observations	39203				168421				147016				168499			
Groups	13828				13948				13908				13948			

Note: all numeric and ordinal variables, both IVs and DVs (except those already binary) were scaled (m = 0, SD = 1). Correlation structure AR (1).

### Health wellbeing

Health-related quality of life and satisfaction with health for CSOs are consistently lower than for non-CSOs. However, the trajectory of both is similar to that experienced by non-CSOs (Table 4).

**Table 4:** Generalised linear mixed-effects model results for health variables.

	Health DV’s							
	SF-6D				Satisfaction with Health			
Fixed Effects	Estimate	*SE*	*t*	*p*	Estimate	*SE*	*t*	*p*
(Intercept)	0.11	0.01	11.27	<.001	0.13	0.01	13.07	<.001
Year	0.09	0.00	29.93	<.001	0.11	0.00	35.19	<.001
CSO—Yes	-0.20	0.04	-5.25	<.001	-0.10	0.04	-2.68	.007
Female	-0.16	0.01	-11.93	<.001	-0.05	0.01	-3.78	<.001
Age	-0.13	0.01	-18.19	<.001	-0.13	0.01	-18.87	<.001
Education	0.05	0.01	6.91	<.001	0.00	0.01	0.59	.555
Household Income	0.19	0.01	27.71	<.001	0.13	0.01	18.69	<.001
Year x CSO	0.03	0.02	1.83	.067	0.03	0.02	1.51	.132
**Random Effects**	St. Dev	Correlation			St. Dev	Correlation		
(Intercept)	0.69	-0.11			0.70	0.01		
Year	0.21				0.24			
Residual	0.62				0.63			
Parameter estimates (phi)	0.13				0.19			
Observations	123170				168651			
Groups	13895				13948			

Note: all numeric and ordinal variables, both IVs and DVs (except those already binary) were scaled (m = 0, SD = 1). Correlation structure AR (1).

### Financial wellbeing

Table 5 indicates that over the 18 years, CSOs have consistently had more financial stressors and lower satisfaction with their finances than non-CSOs. While there was no significant difference in the trajectory of financial stressors between CSOs and non-CSOs, as illustrated in Fig 3 (panel h), the CSOs’ satisfaction with their financial situation does not increase over the years as much as non-CSOs.

**Table 5:** Generalised linear mixed-effects model results for financial wellbeing variables.

	Financial DV’s							
	Financial Stressors				Satisfaction with Finances			
Fixed Effects	Estimate	*SE*	*t*	*p*	Estimate	*SE*	*t*	*p*
(Intercept)	0.04	0.01	4.36	<.001	-0.07	0.01	-7.63	<.001
Year	0.05	0.00	13.98	<.001	-0.10	0.00	-32.02	<.001
CSO—Yes	0.25	0.04	6.68	<.001	-0.22	0.04	-6.18	<.001
Female	0.04	0.01	3.38	.001	0.01	0.01	0.53	.599
Age	-0.21	0.01	-32.54	.000	0.22	0.01	33.99	<.001
Education	0.01	0.01	1.20	.231	0.01	0.01	0.95	.342
Household Income	-0.25	0.01	-37.17	<.001	0.24	0.01	37.35	<.001
Year x CSO	0.00	0.02	-0.08	.937	0.04	0.02	2.08	.038
**Random Effects**	St. Dev	Correlation			St. Dev	Correlation		
(Intercept)	0.65	0.14			0.54	0.20		
Year	0.24				0.21			
Residual	0.70				0.70			
Parameter estimates (phi)	0.23				0.24			
Observations	148585				168575			
Groups	13897				13947			

Note: all numeric and ordinal variables, both IVs and DVs (except those already binary) were scaled (m = 0, SD = 1). Correlation structure AR (1).

## Discussion

We examined the health and wellbeing trajectories of a group of household CSOs in 2018, for the 17 years prior to when it was known from the survey that they were living with someone experiencing a gambling problem. This CSO group was compared to people without a gambling problem in the household at that time. As expected, some social and financial wellbeing outcomes showed a clear temporal effect, declining closer to the time they were known to be impacted by the gambling problem. However, while all measured health and wellbeing outcomes were worse for CSOs, no temporal effects were found with any measured general wellbeing or health variables. We also found around half (49.9%) of the CSO group had also been CSOs three years prior. This is relatively consistent with previous findings [13, 15] and provides further support for our assumption that the likelihood of experiencing impact from another person’s gambling declined with decreasing proximity to the time household gambling problems were measured.

The study identified clear temporal effects associated with social and financial wellbeing. That is, there were significantly different trajectories for CSOs and non-CSOs as proximity to known exposure to household gambling problems decreased. For those without a gambling problem in the household, satisfaction with their financial situation improved as they aged; however, this was not the case in CSOs, whose satisfaction remained reasonably stable over time and lacked improvement. One of the most common gambling-related harms, financial problems can be the first consequence of excessive gambling (Langham et al., 2016; Mathews & Volberg, 2013). Consequently, gambling-related financial problems might offset any normal improvements expected in a person’s financial wellbeing over time. Social wellbeing also appeared to be influenced by gambling problems. For participants not associated with a household gambling problem, social wellbeing appeared relatively stable over time. However, CSOs perceived that social support and their satisfaction with their community deteriorated closer to the point at which it was known they were living with someone with a gambling problem. While both groups reported similar levels of social wellbeing early in the study, the social wellbeing of CSOs had decreased by 2018. This finding supports previous research on social impacts associated with CSOs. CSOs can feel excluded from friends and family and limit social activities due to the fear of being stigmatised because of the gambling behaviour [59], feel shame about staying with the person, or have a fear of being judged [40]. They may be embarrassed because the person who gambles does not attend social events due to time spent gambling, and the CSOs have to attend alone or explain the absence [12]. CSOs might not want to socialise in places with an opportunity to gamble [59]. It might be expected that the social impact is stronger in partners or household CSOs (such as examined in this study) as their social lives may be more interlinked than non-household CSOs such as friends and work colleagues.

Contrary to expectations, in other areas of health and wellbeing, no temporal effects were found. Trajectories of health-related quality of life and satisfaction with health were similar for both CSOs and non-CSOs. However, CSOs reported lower mean values, reporting consistently lower health-related quality of life and satisfaction with health than non-CSOs. These lower means are consistent with cross-sectional studies that tend to find CSOs report impaired physical and mental health [e.g. 17, 20, 60]. Over the period of the study, CSOs reported a higher number of personal, deviant, and financial stressors than non CSOs, however these trajectories did not measurably differ with proximity to the known gambling problem. Life satisfaction stayed relatively steady over time for all participants. This findings fits the theory that people have their own personal level of satisfaction with life [61]. Over time, notwithstanding a few bumps along the way, this will usually return to that person’s ‘normal’ level. Again, the trajectory of CSOs’ satisfaction with life was similar to non-CSOs, while CSOs reported lower mean satisfaction overall. Paterson and colleagues [62] found similar results when they explored the trajectory of life satisfaction in those experiencing a first-hand gambling problem. Their study found those with a serious gambling problem reported consistently lower levels of life satisfaction across 15 years compared to non-problem and at-risk gamblers.

Our findings suggest that some of the lower health and life satisfaction factors commonly associated with CSO’s may not be directly or solely attributable to the gambling problem. While CSOs were found to experience long-standing decrements to their health and reported life satisfaction, these did not change with proximity to exposure to the gambling problem. Instead, it may be that these issues are related to some risk factors for having a gambling problem in the household, or some factor unrelated to gambling. For example, CSOs tend to be younger, less educated, and more likely to have a gambling problem themselves [4, 15, 20, 21, 60]. Additionally, gambling problems are associated with renting in low socioeconomic areas, other addictions, and other mental health challenges [56, 63, 64]. All these factors have their own complex relationships with health and wellbeing [65–67], and may affect the health and life satisfaction of household CSOs.

### Limitations and further research

The principal limitation of the study was that CSO status was only measured in 2018, meaning that full longitudinal modelling of this impact to consequences could not be done. The retrospective analysis undertaken rests on the uncontroversial assumption that proximity to gambling problems is not permanent: given a positive case in 2018, the probability of being a CSO in a prior year tends to decline with increasing time. Nevertheless, the unmeasured variable and the stochastic nature of the temporal relationship inject significant noise into the analysis. Additionally, the sample size and variability within the data may not have provided enough power to identify some smaller effects over-time, while clustering may have yielded anti-conservative tests of significance. Further purpose-designed longitudinal studies will be needed to make firmer conclusions, for example by measuring CSO status across all years and larger samples that enable comparative analyses of CSOs living with gamblers in the problem and at-risk categories of the PGSI. Finally, the analytic sample consisted of CSOs living in the same household as a person with a gambling problem. While an important sub-sample, those impacted by another person’s gambling comprise a much wider group [2, 4, 59]. Ex-partners, for example, comprise a significantly sizeable proportion of people harmed by another’s gambling problem [4] and can experience long-term financial and relational harm [6, 59, 68].

## Conclusions

Overall, the study found a temporal effect between exposure to another person’s gambling problem and negative social and financial wellbeing outcomes for CSOs. This indicates that a gambling problem in the household is likely to directly contribute to decreases in the social and financial wellbeing of other people living in that household. On the other hand, no clear temporal link was found with health, life satisfaction, or the number of stressors experienced by CSOs; that is, there were no significant changes with respect to proximity to the gambling problem. Instead, CSOs reported long-term health and overall wellbeing decrements, which likely preceded exposure to the gambling problem. This might indicate that these decrements may not necessarily be a direct outcome of problem gambling exposure. Instead, they may be related to other risk factors associated with having a gambling problem in the household. However, regardless of what is causing or contributing to health and wellbeing decrements, they should continue to be considered by policy and practice that aims to support CSOs.
